# Dynamics of rectal balloon implant shrinkage in prostate VMAT: Influence on anorectal dose and late rectal complication risk

**DOI:** 10.1007/s00066-017-1222-x

**Published:** 2017-10-16

**Authors:** Ben G. L. Vanneste, Y. van Wijk, L. C. Lutgens, E. J. Van Limbergen, E. N. van Lin, K. van de Beek, P. Lambin, A. L. Hoffmann

**Affiliations:** 1 Department of Radiation Oncology (MAASTRO), GROW – School for Oncology and Developmental Biology, Maastricht University Medical Center+, P.O. Box 3035, 6202 NA Maastricht, The Netherlands; 2 Radiotherapy Bonn-Rhein-Sieg, Troisdorf, Germany; 3 Department of Urology, Maastricht University Medical Center+, Maastricht, The Netherlands; 4 Institute of Radiooncology – OncoRay, Helmholtz-Zentrum Dresden–Rossendorf, Dresden, Germany; 5 Department of Radiotherapy, University Hospital Carl Gustav Carus at the Technische Universität Dresden, Dresden, Germany

**Keywords:** Volumetric modulated arc therapy, Radiotherapy, Rectum, Volume stability, Toxicity, Volumenmodulierte Arc-Therapie, Strahlentherapie, Rektum, Volumenstabilität, Toxizität

## Abstract

**Purpose:**

To assess the effect of a shrinking rectal balloon implant (RBI) on the anorectal dose and complication risk during the course of moderately hypofractionated prostate radiotherapy.

**Methods:**

In 15 patients with localized prostate cancer, an RBI was implanted. A weekly kilovolt cone-beam computed tomography (CBCT) scan was acquired to measure the dynamics of RBI volume and prostate–rectum separation. The absolute anorectal volume encompassed by the 2 Gy equieffective 75 Gy isodose (V_75Gy_) was recalculated as well as the mean anorectal dose. The increase in estimated risk of grade 2–3 late rectal bleeding (LRB) between the start and end of treatment was predicted using nomograms. The observed acute and late toxicities were evaluated.

**Results:**

A significant shrinkage of RBI volumes was observed, with an average volume of 70.4% of baseline at the end of the treatment. Although the prostate–rectum separation significantly decreased over time, it remained at least 1 cm. No significant increase in V_75Gy_ of the anorectum was observed, except in one patient whose RBI had completely deflated in the third week of treatment. No correlation between mean anorectal dose and balloon deflation was found. The increase in predicted LRB risk was not significant, except in the one patient whose RBI completely deflated. The observed toxicities confirmed these findings.

**Conclusions:**

Despite significant decrease in RBI volume the high-dose rectal volume and the predicted LRB risk were unaffected due to a persistent spacing between the prostate and the anterior rectal wall.

## Zusammenfassung

**Zielsetzung:**

Beurteilung der Wirkung eines schrumpfenden rektalen Ballonimplantats (RBI) auf die anorektale Dosis und das Komplikationsrisiko im Verlauf einer mäßig hypofraktionierten Strahlentherapie der Prostata.

**Methoden:**

Ein RBI wurde 15 Patienten mit lokal begrenztem Prostatakarzinom implantiert. Zur Messung der Dynamik des RBI-Volumens und der Prostata-Rektum-Trennung wurde eine wöchentliche Kilovolt-Cone-beam-Computertomographie (CBCT) aufgenommen. Das absolute anorektale Volumen, das von der 2 Gy äquieffektiven 75-Gy-Isodose (V_75Gy_) umfasst wurde, wurde ebenso berechnet wie die mittlere anorektale Dosis. Die Zunahme des geschätzten Risikos für späte rektale Blutungen (LRB) vom Grad 2–3 zwischen Beginn und Ende der Behandlung wurde mit Nomogrammen vorhergesagt. Die beobachteten akuten und späten Toxizitäten wurden ausgewertet.

**Ergebnisse:**

Es wurde eine signifikante Schrumpfung der RBI-Volumina beobachtet, mit einem durchschnittlichen Volumen am Ende der Behandlung von 70,4 % des Baseline-Volumens. Obwohl die Prostata-Rektum-Trennung im Verlauf der Therapie deutlich abnahm, betrug diese durchgehend mindestens 1 cm. Es wurde kein signifikanter V_75Gy_-Anstieg des Anorektums beobachtet, außer bei einem Patienten, dessen RBI in der dritten Behandlungswoche vollständig entleert war. Zwischen der mittleren anorektalen Dosis und der Ballonvolumenabnahme wurde keine Korrelation festgestellt. Die Zunahme des LRB-Risikos war nicht signifikant, außer bei dem Patienten mit vollständig entleertem RBI. Die beobachteten Toxizitäten bestätigten diese Befunde.

**Schlussfolgerung:**

Trotz signifikanter Abnahme des RBI-Volumens waren das Hochdosis-Rektumvolumen und das vorhergesagte LRB-Risiko aufgrund eines anhaltenden Abstands zwischen der Prostata und der anterioren Rektumwand davon nicht nachteilig betroffen.

External beam radiation therapy (EBRT) is an effective curative treatment option for patients with localized adenocarcinoma of the prostate compared to surgery [1]. Increasing radiation dose is associated by increased control; however, this is correlated with an increased potential risk of gastrointestinal (GI) toxicity, with possibly a decrease in the quality of life [2–4]. Despite the development of advanced treatment techniques like intensity-modulated radiation therapy, volumetric arc therapy, and image-guided radiotherapy, sparing of the rectal wall is a prerequisite for safe delivery of high doses to the prostate. This makes the rectum the dominant dose-limiting organ at risk in prostate EBRT. To spare the rectum, artificial spacing material has been used for insertion into the retroprostatic space. Implantable rectum spacers (IRS) separate the anterior rectal wall from the prostate by creating an artificial distance between these organs. As such they reduce the dose exposure to the rectum, consequently decreasing the risk of GI toxicity. Different types of IRS exist, all of which are implanted through a transperineal approach. Hyaluronic acid [5] and collagen implants [6] are physiological compounds made of substances that are naturally present in the human body. Potential side effects for transmission of infectious agents or immunological reactions have been reported [7]. Therefore, commercially available spacers based on polyethylene glycol (PEG) hydrogels [8] and biodegradable saline-filled rectal balloon implants (RBI) [9] have been developed. Recently Wolf et al. [10] compared PEG and RBI spacer technologies in 59 prostate cancer patients undergoing radiation treatment and concluded that the RBI was superior in reducing rectum dose, whereas the PEG hydrogel spacer had a better volume consistency with respect to the duration of treatment. They reported an early and sudden RBI volume decline in 4 out of 16 patients, and an average volume loss of >50% in the remaining 12 patients during the full course of treatment over 8 weeks of a normofractionated radiotherapy regimen comprising 41 fractions.

The aim of our study was to evaluate the RBI volume stability and the dosimetric effect of RBI volume shrinkage on the anorectum and to estimate the 3‑year risk of grade 2–3 late rectal bleeding (LRB) during the course of a moderately hypofractionated EBRT regimen comprising 28 fractions. We tested the hypotheses that despite of an expected RBI volume decrease over time there is no significant increase in the absolute anorectal volume encompassed by the 2 Gy equieffective dose of 75 Gy (V_75Gy_) and in the mean anorectal dose, both of which are considered relevant parameters for predicting LRB. We also tested the hypothesis that there is no correlation between RBI deflation and mean anorectal dose. In addition, we hypothesized that the predicted increase in risk of LRB resulting from the volume decrease of the RBI is insignificant. Furthermore, we postulated that this predicted status quo of LRB risk can be explained by a persistent prostate–rectum separation of at least 1 cm during the whole course of treatment. Finally, we reported the observed acute and—as far as possible—late toxicities.

## Materials and methods

### Patient selection

After approval by the institutional review board (number 14-38-03/09-internal-6335), 15 consecutive prostate cancer patients were prospectively included in this study between June 2015 and March 2016. Patients with a histologically confirmed, localized adenocarcinoma of the prostate were enrolled in this study to receive an RBI (BioProtect Ltd, Israel). All patients had signed an informed consent form. The patient and tumor characteristics are summarized in Table 1. Patients who had been classified as intermediate risk were prescribed additional neoadjuvant hormonal therapy for 6 months [11]. The high-risk patients were offered an additional 1.5 years hormonal therapy as an extension of the 6‑month neoadjuvant therapy after EBRT. All patients underwent magnetic resonance imaging (MRI) to exclude extraprostatic spread. Dorsal extraprostatic disease extension (stage T3a/4) was an exclusion criteria, as well as distant metastatic disease, inflammatory bowel disease and previous pelvic EBRT.

**Table 1:** Patient (*N* = 15) and tumor characteristics

*Age* (years; median [range])	72 [63–77]
*Prognostic risk group* ^*a*^ *:* (no. of patients)	
1 – Low risk	1 (7%)
2 – Intermediate risk	5 (33%)
3 – High risk	9 (60%)

^a^ *Low risk*: no risk factors: PSA <10 ng/ml; Gleason score <7; cT stage <2b; *Intermediate risk:* PSA 10–20 ng/ml and/or Gleason score = 7 or cT stage = 2b/c; *High risk:* PSA >20 ng/ml or Gleason score >7 or cT stage >2b/c

### RBI implantation procedure

An RBI was implanted in these patients between the prostate and the anterior rectal wall 7–10 days prior to the start of EBRT. The injection technique has been previously described in detail [12]. A short general anesthesia is preferred at our department. However, the implantation procedure can be also performed under local or spinal anesthesia. First, four fiducial markers (PolyMark™, CIVCO, Orange City, IA, USA) were implanted intraprostatically for daily position verification. The RBI was implanted transperineally under biplane transrectal ultrasonography guidance. A bubble-free (sterile) saline solution was used to fill and inflate the RBI. The saline solution was mixed with approximately 1.5 cm^3^ iodinated contrast medium to enhance the visualization of the RBI on computed tomography (CT) scans. The injected volume was varied, depending on the volume of the prostate. Because the RBI should not be filled to achieve a prostate–rectum separation larger than 30 mm, we adapted the volume of the RBI to the volume of the prostate: small prostates (<35 ml) do not need the maximum RBI volume to guarantee a prostate–rectum separation of at least 1 cm, which is considered as a conclusive spread [13].

### Target volume definition, dose prescription, and treatment planning

Each patient underwent a CT scan and MRI scan 5–7 days after RBI implantation in supine position with a slice thickness of 3 mm for treatment planning and target volume delineation purposes, respectively. One hour prior to image acquisition, patients were instructed to first empty their bladder, then drink 300 ml of water to have a full bladder, and empty their bowel. No use of laxative was recommended. The CT and MRI scans (balanced turbo field echo sequence with isotropic 0.5 mm in-plane resolution) were coregistered based on the fiducial markers.

**Fig. 1:**
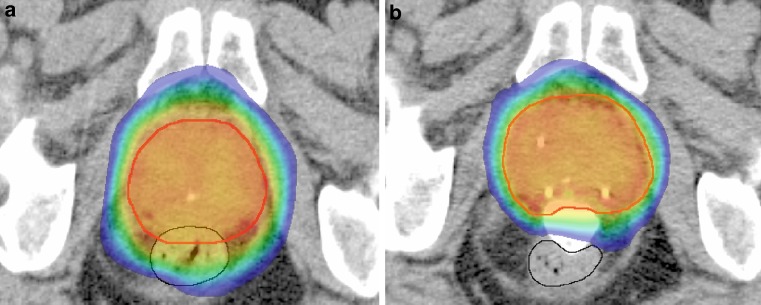
Color-wash isodose distribution projected on an axial CT slice before (**a**) and after RBI implantation (**b**) in the same patient with the planning target volume in *red*. Without RBI (**a**), the high-dose region >80% (*green isodose*) overlaps with the entire ventral part of the rectum (*black line*), whereas with the RBI in situ (**b**) the rectum is exposed to a dose <65% (*blue isodose*)

Delineation of the prostate (CTV = clinical target volume) was performed on the T_2_-weighted MRI scan, while the RBI and the organs at risk were delineated on the CT scan (Fig. 1). In case the CT and MR images showed different prostate shapes and volumes (e. g., due to differences in rectal filling), the MR imaging was repeated. The first planning target volume (PTV1) was constructed according to the institutional protocol by expanding the CTV with 10, 7 and 6 mm in cranial–caudal, anterior–posterior, and left–right direction, respectively. A second PTV (PTV2) was defined as a 5 mm isotropic expansion of the CTV, with exclusion of the anorectum and bladder. All treatment plans were designed for dose delivery with a volumetric modulated arc technique (VMAT) using 10 MV photon beams (Eclipse Version ICD-10, Varian Medical Systems Inc., Palo Alto, CA, USA). The prescribed dose to PTV1 and PTV2 was 65.8 and 70 Gy, in 28 fractions of 2.35 and 2.5 Gy, respectively [14]. With α/β = 3 Gy for late rectal toxicity, the maximum 2 Gy equieffective dose (EQD2_3_) in the anorectum for this type of plan is 77 Gy [15]. No density override for the iodine-containing RBI was performed because it was assumed that the contrast medium that was present in the RBI at the time of CT scanning and treatment planning remained present during treatment delivery. The overall treatment time was 7 weeks, at 4 fractions a week. The dose–volume constraints fulfilled the institutional protocol, which is based on the QUANTEC guidelines [16]. All patients underwent daily X‑ray based position verification and repositioning based on the intraprostatic fiducial markers.

### RBI volume and distance dynamics

To assess the volume stability of the RBI over time, a weekly kilovolt cone beam computed tomography (CBCT) scan was acquired in treatment position prior to irradiation, respectively at fractions 1, 7, 11, 15, 19, 23, and 27. The resulting 105 CBCT scans were imported into the treatment planning system for delineation and subsequent 3D volumetry of the RBI. Furthermore, the minimum distance between the prostate and the anterior rectal wall was measured at midprostate level in anterior–posterior direction. Two observers independently delineated the RBI (RG and BV) and the anorectum (DH and BV). The anorectal structure consists of the rectum and the anal canal. The rectum was delineated from the top of the anal canal up to the rectosigmoid flexure. The anal canal was considered as the distal 3 cm of the anorectum. Six to nine months after RBI implantation, an MRI scan was acquired to evaluate the biodegradability of the RBI.

### Anorectal dose estimation

To assess the dosimetric consequences of RBI shrinkage on the anorectum, the planned dose distribution of the three patients with the largest observed RBI volume reduction was recalculated on the weekly acquired CBCT images, while keeping all planning parameters (e. g., beam arrangement, field size, fluence maps, monitor units) the same as for the initial, CT-based treatment plan. The dose–volume histogram (DVH) for the anorectum obtained from the treatment plan was converted into EQD2_3_ using the Withers formula [17]:

$$\mathrm{EQD}2_{\alpha /\beta }=D\cdot \frac{d+\frac{\alpha }{\beta }}{2+\frac{\alpha }{\beta }},$$

where α/β = 3 Gy for late rectal toxicity, *D* is the total dose, and *d* is the dose per fraction. All dose(–volume) parameters were obtained from the EQD2_3_-converted DVH. The absolute V_75Gy_ of the anorectum was compared between the CT and CBCT plans: in the 3 cases with the largest RBI volume decrease the weekly CBCT images were compared. For the remaining patients, only the CBCT images of the last week (CBCT_27_) were used for comparison of the anorectal V_75Gy_ against the CT-based plan. The EQD2_3_-converted mean anorectal dose was calculated for the planning CT and the final CBCT for all patients.

### Complication risk estimation

To assess the effect of the RBI volume decrease on the 3‑year risk of grade 2–3 LRB, a set of previously published multifactorial nomograms were used [18]. These nomograms use clinical parameters (use of anticoagulants, hormonal therapy, or antihypertensives; pelvic node irradiation; presence of diabetes or hemorrhoids; and a history of pre-RT abdominal surgery), in addition to dosimetric parameters (mean rectal dose and the percentage of the anorectum volume receiving at least an EQD2_3_ of 75 Gy) to predict the risk of late rectal bleeding. The nomograms were applied to the initial treatment plans and the plans performed on the final CBCT images for each patient. The results were used to estimate the largest change in predicted complication risk due to shrinkage of the RBI.

### Observed toxicity assessment

The complications were recorded in terms of Common Terminology Criteria for Adverse Events (Version 4.03) [19]. Acute gastrointestinal (GI) and genitourinary (GU) toxicities were scored in the 2nd, 4th, 6th week of treatment and 3 months after its completion. Late toxicities were scored in the 6th, 9th, and—in case if possible—12th, and 18th months after treatment completion.

### Statistical analysis

The statistical analyses were carried out using the Statistics Toolbox of MATLAB (Version 10.2, The MathWorks, Natick, MA, USA) software. The paired-samples Wilcoxon signed rank test was applied to test for a significant decrease in volume of the RBI on weekly acquired CBCT scans, and for a significant decrease in distance between prostate and rectum. This test was also applied to test for a significant increase in predicted complication risk between the first and last fraction. All statistical tests were one-sided, with *P* < 0.05 considered to be statistically significant.

## Results

### RBI volume and distance stability

**Fig. 2:**
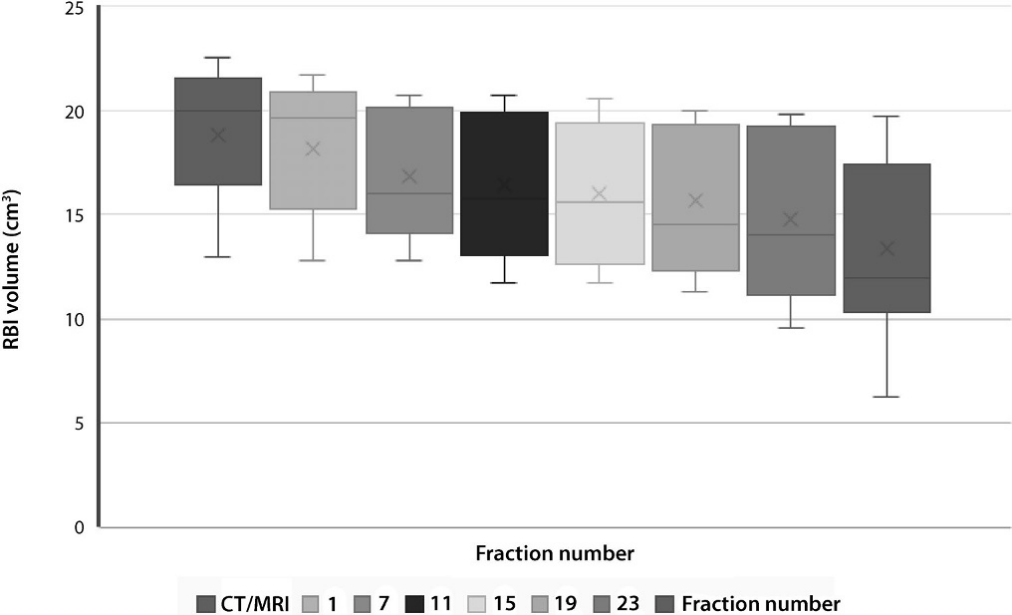
A box and whisker plot of the delineated rectal balloon implant (*RBI*) volumes during a full treatment course of 28 fractions in 13 patients (*grey dashed lines*) observed by weekly cone-beam computed tomography (*CT*) images

The median injected and delineated RBI volumes were 17.0 cm^3^ (range 9–17 cm^3^) and 20.0 cm^3^ (range 12.9–22.6 cm^3^), respectively. Volume differences are explained by the fact that the delineated RBI structure comprises both the injected saline solution and the RBI envelope. Two patients were excluded from the stability analysis because in one patient CBCT scans were missing due to a protocol violation and in another patient the RBI had disappeared on CBCT in the third week of treatment as no contrast medium could be detected. For the remaining 13 patients, the descriptive statistics of the RBI volume dynamics are summarized in Table 2 and depicted in Fig. 2. The median RBI volumes at fractions 1, 15, and 27 were 19.6 (range 12.8–21.7), 15.6 (range 11.7–20.6), and 11.9 cm^3^ (range 6.3–19.8 cm^3^), respectively. The weekly decrease in absolute RBI volume was significant for all time points, with an average volume loss of 29.6% at fraction 27 relative to baseline. The largest relative volume decrease occurred during the first week (i. e., between fraction 1 and 7), and after fraction 19 (Fig. 2). The median volume loss relative to baseline was 25% (range 5.7–54.9%). The descriptive statistics of the prostate–rectum distance dynamics are also summarized in Table 2. Although the weekly decrease of minimum prostate–rectum distance was significant for all time points, this distance remained greater than 1 cm at all times.

**Table 2:** RBI volume and minimum prostate–rectum distance dynamics

	*RBI volume* (cm^3^; median [range])	*Prostate*–*rectum distance* (cm; median [range])
*Planning CT*	20.0 [12.9–22.6]	2.3 [1.9–2.9]
*CBCT 1*	19.6 [12.8–21.7]	2.2 [1.8–2.8]
*CBCT 7*	16.0 [12.8–20.7]	2.0 [1.7–2.5]
*CBCT 11*	15.8 [11.7–20.7]	1.9 [1.6–2.4]
*CBCT 15*	15.6 [11.7–20.6]	1.9 [1.5–2.4]
*CBCT 19*	14.5 [11.3–20.0]	1.9 [1.3–2.4]
*CBCT 23*	14.0 [9.6–19.8]	1.7 [1.1–2.3]
*CBCT 27*	11.9 [6.3–19.8]	1.4 [1.1–2.3]

*RBI* rectal balloon implant, *CBCT* cone-beam computed tomography

### RBI resorption

Six to nine months after completion of EBRT a residual rest of the RBI envelope was visible on T_2_-weighted MRI in only 1 out of 15 (7%) patients; no contrast medium or saline solution could be observed. Neither space-occupying effects nor complications (infections, perforations, fibrosis) were observed in any of the patients.

### Anorectal dosimetry

**Fig. 3:**
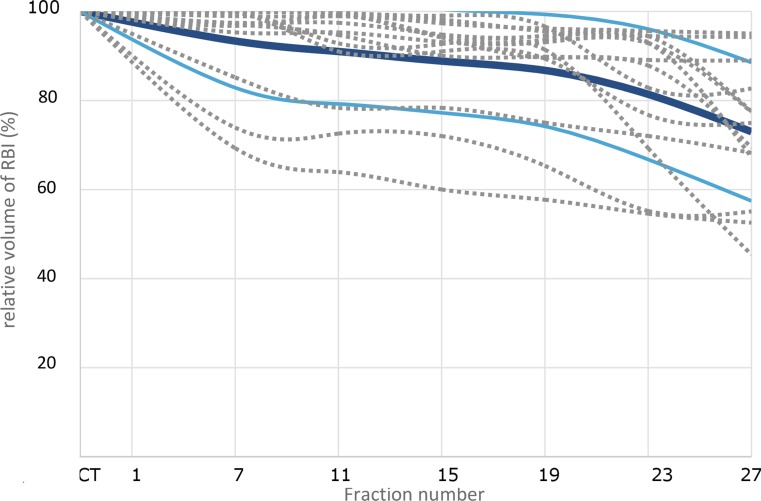
Relative volume dynamics of rectal balloon implant during a full treatment course of 28 fractions in 13 patients (*grey dashed lines*) observed by weekly cone-beam computed tomography (*CT*) images. The mean values (*dark blue*) with the standard deviations (*light blue*) are presented

In the patient whose RBI had disappeared on the CBCT scan in the third week of treatment, the V_75Gy_ of the anorectum significantly increased from 0 to 4.3 and 5.9 cm^3^ at fraction 1, 11, and 27, respectively (patient 9, Fig. 3.) In the other two patients (numbers 5 and 12) exhibiting the largest RBI volume decrease (45 and 52% of the original volume, respectively) a significant increase in absolute V_75Gy_ of the anorectum was only observed in the CBCT scan of fraction 23 (patients 5 and 12, Fig. 3). Furthermore, the RBI deflation did not lead to significant increase in V_75Gy_ (*p* = 0.577). Among the remaining patients there was only one patient who showed an increase of V_75Gy_ from 0 cm^3^ on the CT plan to 3.6 cm^3^ on the CBCT_27_ plan (Table 3). In 2 out of 11 patients, the V_75Gy_ remained 0 for both the CT and CBCT_27_ scans. In 6 patients, even a slight decrease of V_75Gy_ was determined. The increase in V_75Gy_ for all patients except the one of whom the RBI disappeared was not significant (*P* = 0.57). The increase in mean anorectal dose was significant (*P* = 0.02); however, no correlation was found between the mean anorectal dose and the volume of the RBI (*P* = 0.3). The correlation between the anorectal volume and the anorectal mean dose was significant (*P* = 0.005).

**Table 3:** Descriptive statistics for treatment plans based on the planning CT scan and the CBCT scan of fraction 27

	*Planning CT* median [range]	*CBCT*_*27*_ median [range]
*Rectal volume (cc)*	88.7 [56.1–187.4]	68.65 [45.3–200.8]
*Mean rectal dose (Gy)*	26.2 [10.9–34.6]	33.9 [19.3–38.7]
V_75Gy_ * (cc)*	0.1 [0.0–1.6]	0.7 [0.0–5.9]
*Complication risk (%)*	4.1 [3.8–11.9]	4.5 [3.9–11.6]

*CT* computed tomography, *CBCT* cone-beam computed tomography, *V*_*75Gy*_ volume receiving at least a 2 Gy equieffective dose (EQD2_3_) of 75 Gy, *Mean rectal dose* mean EQD2_3_ dose in the anorectum

### Risk of late rectal bleeding

**Fig. 4:**
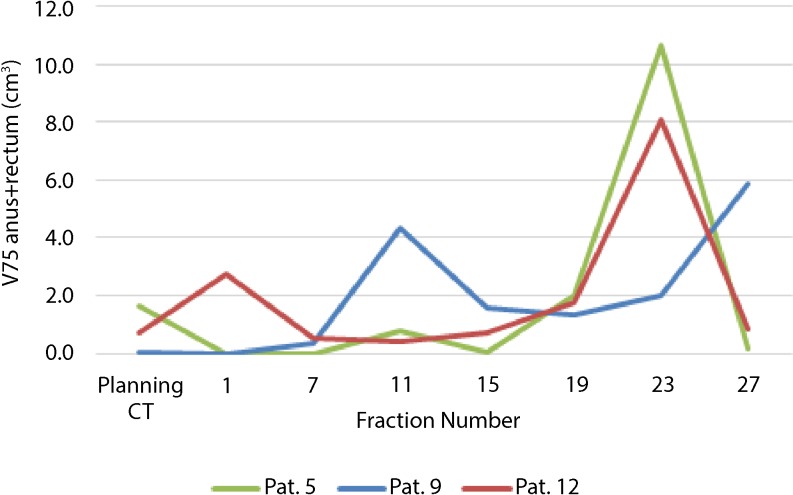
Dynamics of estimated absolute anorectal volume encompassed by the biological equivalent 75 Gy isodose during a full treatment course of 3 patients (numbers 5, 9, 12) exhibiting the largest volume decrease of the implanted rectal balloon. Patient 9 had the complete rectal balloon implant (*RBI*) shrinkage on cone-beam computed tomography (*CBCT*) of fraction 11: the V_75Gy_ increased significantly. In the other two patients (5 and 12) a significant increase in absolute V_75Gy_ of the anorectum was observed in only one CBCT scan (fraction 23) over the total treatment course

**Fig. 5:**
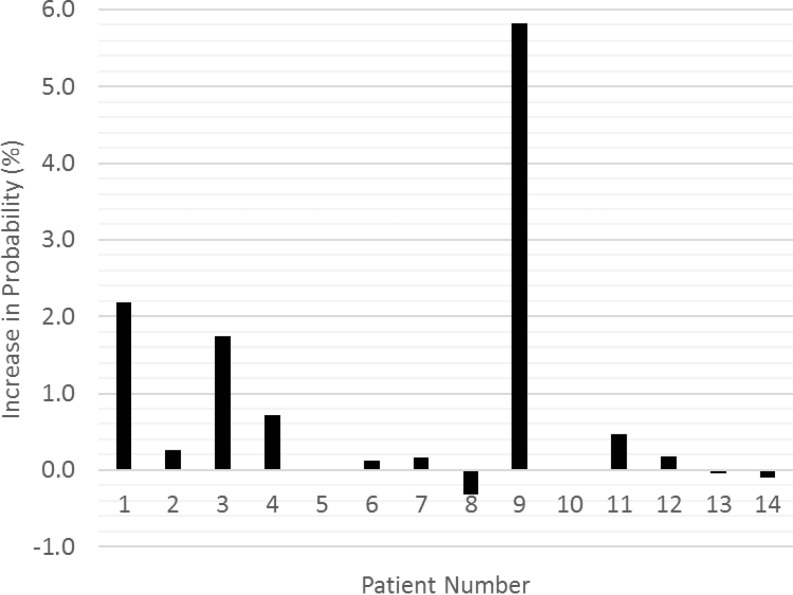
Increase in predicted probability (percentage points) of late rectal bleeding between the planning computed tomography (*CT*) and the last cone-beam CT (*CBCT*) scan (fraction 27) for each of the patients

In the patient whose RBI had completely deflated in the third week of treatment (patient 9), the risk of LRB was predicted to increase from 4.0 to 9.8%. For the two patients with the largest observed RBI shrinkage (patients 5 and 12) the predicted increase in LRB risk was 0 and 0.2%, respectively (Fig. 4). The largest increase in complication risk predicted for the remaining patients was 3.4%, resulting from a RBI volume decrease of 32%. The difference between the predicted risk of LRB at the start of treatment and the end of treatment, excluding the patient with the deflated RBI, was not significant (*P* = 0.07) (Fig. 5).

### Observed toxicities

No acute grade 3 or 4 toxicities were reported during treatment or 3 months after completion. Overall, 5 patients (33%) experienced no toxicity, 6 patients (40%) had grade 1 GU toxicities, and 4 patients (27%) had grade 2 GU toxicities. During the course of therapy, in 4 patients (27%) grade 1 GI toxicity was observed, but no grade 2 or more acute GI toxicities.

No late grade 3 GI toxicity has been reported. Late grade 2 GI toxicity was observed in 1 patient: the rectal bleeding started at 9 months after radiation. This was the patient whose RBI had completely deflated in the third week of treatment. Grade 1 GI and GU late toxicities were reported in 2 patients.

## Discussion

This study provides the first evaluation of RBI volume stability that is based on weekly CBCT measurements during the full course of EBRT. As previously reported in literature, an RBI volume decrease was expected over time. We analyzed the dosimetric consequences of this phenomenon and predicted the increase in risk of 3‑years grade 2–3 late rectal bleeding resulting from a shrinking RBI to assess its potential clinical impact. Moreover, the observed acute and early late toxicities were adjusted.

GI toxicity is the major treatment-related side effect in prostate cancer radiotherapy: the rates of acute and chronic grade ≥2 rectal toxicity has increased by dose-escalated EBRT (up to a dose of 78 Gy) compared with lower doses (e. g., 68 Gy) from 3 to 20% and from 5 to 21%, respectively [20–24]. Therefore, it is advantageous to push the rectal wall out of high-dose regions by implantation of an IRS device [25, 26]. So far, most studies in the literature have reported on the use of a PEG hydrogel as an IRS [27–31]. The RBI has some practical advantages [9]. First of all postimplant correction of the RBI position is possible; if the RBI is dispositioned, it can be easily deflated and replaced, whereas liquid spacers (PEG hydrogels, hyaluronic acid, human collagen) do not permit any correction once being injected [9, 12]. In addition, a chemical reaction is required to occur in PEG hydrogels, which limits the implantation time. Furthermore, since the RBI inflates to a predetermined and predictable shape, the learning curve to obtain an adequate implant is less steep than for PEG hydrogels. In addition, due to the defined shape and homogenous hypodense signal, the RBI has an excellent CT visibility, which is most advantageous for treatment planning and CBCT-based evaluations. Besides, some amount of iodine contrast can be added to the saline to enhance the visualization of the RBI on CT and CBCT scans (Fig. 1). Moreover, because the RBI is a closed system, there is no risk of air or hydrogel injection into vessels.

Nevertheless, disadvantages of the RBI have also been reported. Recently Wolf et al. [10] described an early deflation effect. They reported an average volume loss of >50% during a full treatment course of 37–41 fractions (8 weeks). The volumes they estimated were mainly based on measurements of the diameters of the RBI on two orthogonal X‑ray images and calculations by the volume formula for an ellipsoid cylinder. The measurements were only performed on CBCT scans of fractions 20 and 38. Data on the dynamics of the prostate–rectum separation over time are missing in their study. Wolf et al. only recalculated one treatment plan on a CBCT scan for a single patient whose RBI showed a significant volume loss of 58%, and reported an increase of 9.2 cm^2^ for the rectum volume encompassed by the 95% isodose. We measured 3D volume changes on weekly CBCT scans and observed that a prostate–rectum separation of at least 1 cm is maintained during the full treatment course, except in the patient where the RBI deflated. The persistent spread of at least 1 cm means that also for other RT techniques like 3D conventional EBRT, intensity-modulated radiotherapy, or proton therapy, the sustained spread is considered as enough for protecting the anorectal structures [13].

By evaluating the dose on the anorectal structure, we observed that the V_75Gy_ of the anorectum steadily increased only in one patient whose RBI disappeared completely in the third week of treatment. In the 2 remaining patients where the RBI shrank most, the V_75Gy_ changed significantly in only two CBCT scans during the whole treatment course, but not in the last CBCT scan. This effect was caused by a difference in rectal filling (inclusion of gas bubbles and/or stool). The distance between the prostate and anorectum was still enlarged due to the presence of the RBI. However, the distance between the prostate and anorectum at the level of the cranial part of the rectum (above the RBI) could decrease incrementally. This is especially the case when the rectal filling is increased dramatically, and when seminal vesicles are irradiated. In this situation, the cranial part of the rectum above the RBI anorectum could receive a higher dose. Furthermore, the RBI could exert pressure on the rectum and thereby decrease the rectal volume being exposed to an intermediate and a high dose and increase the volume being exposed to low doses, with unknown clinical consequences. To reduce the low- and intermediate-dose levels, a different treatment planning technique using either an arc technique with an avoidance region near the rectum or using strictly anterior and lateral beams would be required.

By applying multifactorial nomograms on the initial treatment plan and on the final treatment plan, the predicted increase in risk of late rectal bleeding was analyzed. This showed that for the patient with the deflated RBI, the risk considerably increased, emphasizing the effect of the device. For the patient whose RBI had completely deflated, a significant increase in the risk of late rectal bleeding was predicted by the nomogram (Fig. 5). This was confirmed by the late rectal bleeding event 9 months after treatment. For the remaining patients, there was no significant increase in predicted complication risk, suggesting that the decrease in RBI volume has little impact on the effectiveness of the RBI. Some fluctuations in predicted toxicity risk can be seen, but these are likely also due to the rectal filling. Indeed, besides the distance between the rectal wall and the prostate, the rectum size could be a possible predictor of GI toxicity [32–35].

A previous study reported a sudden complete deflation of the RBI in 4 out of 16 patients three weeks after implantation [10]. In our series, one patient experienced such a sudden complete deflation three weeks after implantation. A possible explanation was a nonoptimal positioning: the RBI was positioned more caudally than the others, with the tip of the RBI in the pelvic muscle. Because of this positioning, an excessive force was required to inflate the RBI, which could have damaged the sealing mechanism. Another possible explanation of early deflation could be an excessive filling of the RBI (i. e., prostate–rectum separation larger than 30 mm) with bursting and consequent loss of function: each RBI is handmade, and one has to be sure not to exceed the maximum volume allowed that is indicated on the label of the product.

A limitation of this study is the small number of patients included. As this was a feasibility study, only 15 patients were included. Furthermore, there was no prior consensus on the level and window settings of the CBCT scans, which might have influenced the volumetric results. In addition, the CBCT scans were acquired at different time points and, hence, revealed different bladder and rectum filling, thus, adding extra uncertainty to the comparison performed. Furthermore, the nomograms used are not validated for patients treated with a RBI. More research is needed in larger patient cohorts to obtain more evidence. Finally, we evaluated weekly time points, and not daily, which later could be more representative for the whole treatment.

The RBI was successfully implanted in all 15 patients. The mean RBI volumes revealed an average volume of 70.4% of baseline at the end of treatment. Despite the weekly RBI shrinkage to be significant, neither significant increase in absolute V_75Gy_ of the anorectum nor in predicted LRB risk were observed over the full treatment course of our moderately hypofractionated EBRT regimen. Although the minimum prostate–rectum distance showed a significant decrease, it was at least 1 cm during the full treatment course, indicating that such spacing is sufficient to reduce the anorectal V_75Gy_ of a treatment plan delivered by a volumetric modulated arc technique. In patients experiencing a complete deflation of the RBI, the absolute V_75Gy_ of the anorectum is expected to increase significantly, and so is the predicted risk of late rectal bleeding, and the observed toxicity. We advise to acquire imaging by CBCT scans at regular times during the course of treatment to assess deflation dynamics of the RBI. Only when the prostate–rectum distance decreases to less than 1 cm is a treatment plan adaptation recommended.
